# Impact of Continuous Positive Airway Pressure Treatment on Left Ventricular Ejection Fraction in Patients with Obstructive Sleep Apnea: A Meta-Analysis of Randomized Controlled Trials

**DOI:** 10.1371/journal.pone.0062298

**Published:** 2013-05-01

**Authors:** Hao Sun, Jingpu Shi, Min Li, Xin Chen

**Affiliations:** Department of Clinical Epidemiology, Institute of Cardiovascular Diseases and Center of Evidence Based Medicine, The First Affiliated Hospital, China Medical University, Shenyang, China; Uppsala Clinical Research Center, Sweden

## Abstract

**Background:**

It has been known for a long time that obstructive sleep apnea (OSA) is associated with a decreased left ventricular ejection fraction (LVEF). Continuous positive airway pressure (CPAP) is the gold standard treatment for OSA; however, it is unknown whether or not CPAP treatment will improve the LVEF. The aim of the current study was to assess whether or not CPAP treatment improves the LVEF. A meta-analysis was conducted to determine the effect of CPAP treatment on the LVEF among patients with OSA.

**Methods:**

A literature search of PubMed, the Web of Science, and Cochrane Collaboration’s database were utilized to identify eligible reports for this trial. Ten randomized controlled trails were examined and the meta-analysis was performed using STATA 11.

**Results:**

A significant improvement in the LVEF was observed after CPAP treatment (weighted mean difference(WMD) = 3.59, 95% CI = 1.74–5.44; P<0.001). Subgroup analysis revealed that patients with OSA and heart failure had a significant improvement in the LVEF after CPAP treatment (WMD = 5.18, 95% CI = 3.27–7.08; P<0.001); however, the LVEF of patients with OSA only increased 1.11% and there was no statistical significance (WMD = 1.11, 95% CI = −1.13–3.35; P = 0.331). Furthermore, based on univariate meta-regression analysis, only the baseline AHI had a statistically significant correlation with the LVEF.

**Conclusions:**

Our meta-analysis supports the notion that CPAP may improve the LVEF among patients with OSA.

## Introduction

Obstructive sleep apnea (OSA) is a highly prevalent disease that is characterized by upper airway obstruction that can lead to repetitive episodes of apnea and hypopnea during sleep. An epidemiologic survey has estimated that 2% and 4% of middle-aged women and men are affected by OSA, respectively [1]. Some research has also shown that OSA is an independent risk factor for cardiovascular disease [2]–[3].

Continuous positive airway pressure (CPAP) is considered an established treatment for OSA, although contradictory conclusions regarding the effect of CPAP on left ventricular (LV) function in patients with OSA exists; some studies have shown that LV function is improved [4]–[6], and other studies have demonstrated that LV function is unchanged [7]–[8]. Therefore, measurement of the left ventricular ejection fraction (LVEF) is a significant parameter to evaluate LV systolic function, which is a well-established clinical parameter that has essential diagnostic, therapeutic, and prognostic implications, particularly in the setting of heart failure (HF) [9]–[11]. Recent studies have suggested that patients with OSA have a change in the LVEF after CPAP treatment; however, the conclusions are inconclusive. A community-based study showed that CPAP improves LVEF [12]. Khayat [13] showed that LVEF did not change significantly in OSA patients treated with CPAP. In contrast, the sample sizes of the relevant articles were generally small, and thus inadequate to draw robust conclusions. The purpose of this meta-analysis was to quantitatively evaluate improvement in LV function after CPAP therapy based on measurement of LVEF.

## Methods

This meta-analysis included randomized controlled trials which reported data involving the LVEF of OSA patients before and after CPAP treatment. This study was conducted in accordance with the ‘preferred reporting items for systematic reviews and meta-analyses’ (PRISMA) guidelines. No protocol exists for this meta-analysis.

### Search Strategy

A computerized search was conducted on the following bibliographic databases from January 1990 to November 2012: PubMed; the Web of Science; and the Cochrane Collaboration Database. The free text used for search purposes was ‘obstructive sleep apnea OR obstructive sleep hypopnea’ AND ‘left ventricular ejection fraction OR LVEF’ AND ‘continuous positive airway pressure OR CPAP’. No language or other restrictions were used in the search. Moreover, the reference lists of selected articles and review articles were manually searched.

### Study Selection

The purpose of the study was to identify all randomized controlled trials (RCTs) that reported the effect of CPAP treatment on the LVEF. The identified studies which fulfilled the following predetermined inclusion criteria: (1) the study populations were limited to adults with OSA diagnosed by polysomnography based on an apnea-hypopnea index (AHI) ≥5 events/h; (2) the study populations were limited to patients without central sleep apnea (CSA); (3) the studies included LVEF measurements before and after CPAP treatment and a control group; (4) the intervention was an application of CPAP therapy and the period of follow-up was ≥4 weeks; and (5) the studies consisted of adequate data to perform a meta-analysis.

If the study did not refer to OSA patients or did not report measurements of LVEF while utilizing CPAP, the study was excluded. Two researchers extracted, analyzed, and assessed the quality of the individual studies. If there was an apparent disagreement, a third reviewer was consulted.

### Data Extraction

The following information was abstracted and evaluated independently by two investigators, including the first author’s name, year of publication, country of the study, study design (crossover or parallel), type of control group, number of subjects in each group, duration of follow-up, inclusion/exclusion criteria, gender distribution, mean age, mean body index (BMI), baseline post-treatment AHI, mean daily duration of CPAP therapy, baseline SBP and DBP, and baseline LVEF.

### Quality Assessment

The Jadad scale for RCTs was utilized to evaluate the quality of the studies. All studies were assessed by two researchers. A study was considered high quality if graded with ≥3 scores on Jadad scale. If there were any disagreements between researchers, different opinions were discussed.

### Statistical Analysis

A meta-analysis was conducted using Stata 11 for Windows (Stata, College Station, TX, USA). The pooled estimate of the weighted mean difference (WMD) and 95% CI were calculated, as the outcome measurements were the same for each analysis. The fixed or random effects model was used for non-heterogeneous or heterogeneous data, respectively, as appropriate. Heterogeneity between the results of different studies was examined using a chi-square test (a P-value <0.1 was considered statistically significant) and *I* ^2^ tests (*I* ^2^>50%, significant heterogeneity; *I* ^2^<25%, insignificant heterogeneity) [14]. Furthermore, to explore the possible sources of heterogeneity in CPAP treatment effects, sensitive analysis, subgroup analysis, and meta-regression were conducted. Publication bias was assessed by Begg’s correlation and Egger’s regression [15]–[16].

## Results

### Search Results

Sixty-two full texts were initially searched; the abstracts of all the studies were reviewed and 35 studies were judged to be potentially relevant. Among the 35 studies, 4 review articles and letters were excluded. There were 12 RCTs that were considered appropriate for analysis. One of the 12 studies was subsequently excluded because it did not report the value of LVEF after treatment and another study was also exclude because the patients in this study were not properly randomized into groups. The detailed steps of the literature search are shown in **Figure S1**.

### Characteristics of the Eligible Studies

The 10 remaining trials researched a total of 259 patients. The baseline characteristics of the 10 studies are shown in **Table 1**. Moreover, the characteristics of the populations are shown in **Table 2**. Of the 10 studies, 3 studies were cross-over and the remaining studies were parallel in design. The patients of four studies were OSA patients and the other patients were OSA patients with HF.

**Table 1:** Characteristics of 10 studies included in the meta-analysis.

Author	Year	Country	Study design	Subjects	Control group	Sample (treatment/control)	Follow time (week)	Daily duration (h)	Jadad score
Nguyen *et al*. [17]	2010	America	parallel	OSA	placebo	10/10	12	5.0	3
Gilman *et al*. [18]	2008	Canada	parallel	OSA+HF	blank	12/7	4	6.3	3
Hoekema *et al*. [19]	2008	Netherlands	parallel	OSA	oral appliances	12/13	8	6.9	3
Egea *et al*. [20]	2008	Spain	parallel	OSA+HF	placebo	20/25	12	NR	3
Smith *et al*. [21]	2007	England	crossover	OSA+HF	placebo	12/11	12	3.6	4
Arias *et al*. [22]	2006	Spain	crossover	OSA	placebo	10/11	12	NR	4
Usui *et al*. [23]	2005	Canada	parallel	OSA+HF	blank	8/9	4	6	3
Arias *et al*. [24]	2005	Spain	crossover	OSA	placebo	12/13	12	NR	3
Mansfield *et al*. [25]	2004	Australia	parallel	OSA+HF	blank	19/21	12	5.6	3
Kaneko *et al*. [26]	2003	Canada	parallel	OSA+HF	blank	12/12	4	6.2	3

OSA: obstructive sleep apnea; HF: heart failure; NR: not reported.

**Table 2:** The patient characteristics of the 10 included studies.

Author	Year	Mean Age	% of males	BMI	AHI (events/h)	SBP (mmHg)	DBP (mmHg)	LVEF (%)
Nguyen *et al*. [17]	2010	53.4	90.0	29.8	35.2	124.0	77.1	68.2
Gilman *et al*. [18]	2008	57.2	89.5	30.2	34.1	NR	NR	27.9
Hoekema *et al*. [19]	2008	49.7	89.3	33.3	52.2	149.0	93.0	57.8
Egea *et al*. [20]	2008	63.4	93.3	31.0	41.9	124.6	75.5	27.9
Smith *et al*. [21]	2007	61.0	88.5	31.0	36.0	NR	NR	29.0
Arias *et al*. [22]	2006	51.0	95.7	30.9	44.1	127.0	79.0	66.8
Usui *et al*. [23]	2005	53.5	88.2	30.6	40.4	140.6	68.6	30.2
Arias *et al*. [24]	2005	52.0	100.0	30.5	44.0	126.0	79.0	67.3
Mansfield *et al*. [25]	2004	57.4	94.5	35.4	28.2	NR	NR	35.5
Kaneko *et al*. [26]	2003	55.6	87.5	31.6	41.5	127.0	61.0	26.6

BMI: body mass index; AHI: apnea-hypopnea index; SBP: systolic blood pressure; DBP: diastolic blood pressure; LVEF: left ventricular ejection fraction; NR: not reported.

### Pooled Analysis

According to the baseline data, the characteristics of all the studies were comparable; the LVEF measurements after CPAP treatments were pooled. The LVEF data of three crossover studies were all extracted from the first step and there were few carryover effects. Hence, we performed the analysis, including the three crossover studies. Because all of the OSA patients with heart failure took drug therapy in the treatment or control group and the drug therapy did not have a significant impact on LVEF, the data were also taken into consideration.

A significant improvement in LVEF was observed after CPAP treatment (WMD = 3.59, 95% CI = 1.74–5.44; P<0.001). Significant heterogeneity (P<0.001; *I* ^2^ = 78.9%) existed. We also performed a specific meta-analysis of studies that excluded the Hoekema’s study [19], which used an oral appliance as the control group. The results showed that CPAP therapy improves the LVEF (WMD = 4.12, 95% CI = 2.31–5.93; P<0.001; **Figures S2** **–** **S3**).

### Meta-regression Analysis

The detailed results of meta-regression analysis are presented in **Table 3**. Based on the univariate meta-regression analysis, the dependent variables were the WMD and the covariates included the publication year, duration of CPAP use, mean age of the patients, proportion of males, baseline BMI, baseline AHI, baseline ESS, baseline SBP, baseline DBP, the usage time during the day, and the baseline LVEF. Only the baseline AHI had a statistically significant correlation with the LVEF.

**Table 3:** The results of meta-regression.

Meta-regression variables	N	exp(b)	*p* value
Publication year	10	0.79	0.654
Duration of CPAP use	10	0.89	0.714
Mean age	10	1.35	0.191
% proportion of males	10	0.87	0.617
BMI of baseline	10	1.48	0.514
AHI of baseline	10	0.72	0.002
ESS of baseline	5	0.56	0.439
SBP of baseline	7	0.90	0.424
DBP of baseline	7	0.85	0.122
Usage time of a day	7	0.47	0.667
LVEF of baseline	10	0.92	0.110

BMI: body mass index, AHI: apnea/hypopnea index, ESS: Epworth sleepiness score, SBP: systolic blood pressure DBP: diastolic blood pressure LVEF: left ventricular ejection fraction.

### Subgroup Analysis

Because the effectiveness of CPAP treatment can be influenced by many factors, we performed a subgroup analysis. **Table 4** shows the details. Subgroup analysis revealed that OSA patients with heart failure had a significant improvement in LVEF after CPAP treatment (WMD = 5.18, 95% CI = 3.27–7.08; P<0.001); however, after CPAP treatment, the LVEF of the patients with OSA alone increased 1.11% (WMD = 1.11, 95% CI = −1.13–3.35; P<0.331; **Figure S4)**.

**Table 4:** The results of subgroup analysis.

Subgroup	No. ofstudy	Heterogeneity			WMD	
		*P*	*I* ^2^	WMD	95%CI	*P*
Age						
≤60	8	<0.001	83.7%	3.65	1.08∼6.24	0.005
>60	2	0.306	44.4%	4.15	2.59∼5.71	<0.001
Patients						
OSA without HF	4	0.176	39.3%	1.11	−1.13∼3.35	0.331
OSA with HF	6	0.002	74.0%	5.18	3.27∼7.08	<0.001
Design						
parallel	7	<0.001	79.6%	4.67	2.67∼6.67	<0.001
crossover	3	0.957	0.0%	0.73	−1.30∼2.75	0.482
Control						
placebo	5	0.290	63.1%	2.63	0.33∼4.94	0.025
blank	4	0.013	72.0%	5.93	3.29∼8.56	<0.001
oral appliance	1	_	_	−0.80	−4.19∼2.59	0.644
Duration(week)						
4	3	0.457	0.0%	4.21	2.54∼5.87	<0.001
8	1	_	_	−0.80	−4.19∼2.59	0.640
12	6	<0.001	84.8%	3.70	1.25∼6.15	0.043
Sample size						
≤30	8	0.047	50.9%	2.52	0.58∼4.45	0.011
>30	2	<0.001	93.1%	5.87	2.73∼9.00	<0.001
AHI(events/h)						
≤40	4	0.312	15.9%	6.70	4.61∼8.79	<0.001
>40	6	0.004	71.2%	2.59	0.74∼4.47	0.006

OSA: obstructive sleep apnea; HF: heart failure; AHI: apnea-hypopnea index.

### Sensitivity Analysis

**Figure S5** shows that the pooled effect estimates were positive in spite of omitting patients from the studies, which indicated that there was no significant trend and the overall results were not influenced by an individual study.

### Publication Bias

The publication bias was not considered significant. Neither the Egger’s regression asymmetry test (P = 0.440) nor the Begg’s adjusted rank correlation test (P = 0.721) showed publication bias.

## Discussion

This is the first meta-analysis of RCTs to determine the impact of treating OSA on LVEF. Our meta-analysis has quantitatively assessed the change in LVEF after CPAP therapy in OSA patients. The findings of the present meta-analysis, comprised of 259 patients, suggested that CPAP might be an effective intervention for improving the LVEF in patients with OSA. The results indicated a 3.59% increase in the LVEF after CPAP therapy. Moreover, in a subsequent meta-analysis involving OSA patients with HF, we found that CPAP treatment was associated with a 5.18% improvement in the LVEF.

The present study is robust because the prospective design should eliminate selection and recall bias [27]. Furthermore, all of the 10 studies included in our meta-analysis were RCTs, which were based on most ideal evidence and all of which were ≥3 scales by the Jadad scale. There was no evidence of publication bias and our sensitivity analysis showed no change in the statistical significance of the pooled estimate. Therefore, the outcome of present study was convincing.

There are several factors that could increase cardiovascular morbidity and mortality in OSA patients, such as systemic hypertension, pulmonary hypertension, cardiac systolic and diastolic dysfunction, and cardiac ischemia [28]. In a large epidemiologic study, OSA was independently associated with heart failure [29] and occurred frequently in HF clinical groups [30]–[31]. During obstructive episodes, respiratory efforts decrease intrathoracic pressure, increase left ventricular transmural pressure, and increase left ventricular afterload and cardiac metabolic demand. In addition, augmentation of venous return to the right heart may result in a leftward septal shift (ventricular interdependence) and reduced left ventricular preload and stroke volume [32], which may decrease the LVEF. Alchanatis et al. [33] reported a lower LVEF in an OSA population (53±7%) compared with healthy subjects (61±6%).

In a subgroup analysis, the LVEF of the OSA patients with HF increased significantly after CPAP treatment. Numerous experimental observations were performed to look at the effects of CPAP treatment on various cardiac functions and showed beneficial effects [12], [20]. Most of the observations included patients with OSA and HF. The exact mechanisms involved in which CPAP treatment affects LVEF in HF are not yet completely understood. CPAP treatment may have a positive impact on LV systolic remodeling parameters by reducing the blood pressure, hypoxia, rapid intrathoracic pressure changes, and secondary hemodynamic disturbances [34]. CPAP treatment could also play an important role in improving LV systolic function, hemodynamics, subendocardial ischemia, and the level of oxyhemoglobin [35]. However, Lindsay et al. [36] suggested that the potential therapeutic benefits of CPAP in HF is achieved by alleviation OSA rather than improvement in cardiac function because the CPAP improved daytime sleepiness, but not other subjective or objective measures of HF severity. For this reason, more studies of OSA patients with HF are needed to determine whether or not CPAP treatment may alleviate the symptoms of HF and whether or not CPAP may be used as one approach to treat HF.

Interestingly, the LVEF increased 4.94% after 4 weeks of CPAP treatment and 2.96% after 12 weeks of CPAP treatment, which differed from what we previously thought. Ferrier et al. [37] also demonstrated that the magnitude of increase in the LVEF was 5.1% after 3 months, which was lower than the 9% improvement reported after 1 month [38]. Ferrier et al. [37] explained this finding by differences in study patient characteristics, duration or CPAP usage, and because the long-term acceptance of CPAP treatment was relatively low and may potentially limit effectiveness.

Although our statistical analysis showed that publication bias did not exist, the capacity to detect publication bias was reduced when the meta-analysis was based on a limited number of studies which could not be excluded [15]–[16]. Additionally, most of the studies had a small sample size and primarily included elderly and overweight male subjects; however, several studies have shown that one-third of OSA patients are females [39]–[40]. As a result, it would be of interest to determine whether or not these findings also apply to females. In addition, the styles of the control group were different. Although all of the patients with HF in the placebo group took HF drug therapy which did not significantly impact on the LVEF, it may have affected our results. Another limitation of the current study is the confounding factors of CPAP therapy in patients with OSA, which were complex and diverse, however, our review only included some of the confounding factors, and those unanalyzed factors that were inconsistent with our results might inevitably affect the present results.

According to our meta-analysis, we would like to suggest further recommendations about relevant studies. To extend our results to all patients in a more meaningful manner, additional trials with female OSA patients should be conducted to determine the influence of CPAP on females. In addition, multi-center studies and large sample sizes are needed. Our meta-analysis has illustrated that the LVEF of OSA patients with HF increased significantly after CPAP treatment; however, whether or not CPAP can be used to treat HF is unknown. Therefore, additional trials, including patients with HF alone, should be conducted. Although there are some aspects that should be improved, we considered our study was robust enough to show that CPAP treatment might increase LVEF in patients with OSA at present.

In conclusion, this meta-analysis may provide support for a favorable effect of CPAP treatment on increasing LVEF among patients with OSA. A decrease in the LVEF is one of the well-established indicators of HF, with its clinical use growing in popularity. Therefore, the LVEF might also be considered a useful tool in assessing the efficacy of CPAP treatment in reducing cardiovascular risk in patients beyond the prevention of apneic episodes.

## Supporting Information

Figure S1 **Flow diagram of the literature search.**
(TIF)

Figure S2 **Forest plot presenting the meta-analysis for the effect of CPAP treatment on LVEF.** WMD: weighted mean difference; CI: confidence intervals.
(TIF)

Figure S3 **Forest plot presenting the meta-analysis for the effect of CPAP treatment on LVEF except Hoekema’s study.** WMD: weighted mean difference; CI: confidence intervals.
(TIF)

Figure S4 **Forest plot presenting the meta-analysis for the effect of CPAP treatment on LVEF which were categorized by different patients.** WMD: weighted mean difference; CI: confidence intervals.
(TIF)

Figure S5 **Sensitivity analysis of selected studies.** CI: confidence intervals.
(TIF)

Checklist S1 **PRISMA Checklist of this meta-analysis.**
(DOC)
