# Supplementary figures and images for: Impact of Continuous Positive Airway Pressure Treatment on Left Ventricular Ejection Fraction in Patients with Obstructive Sleep Apnea: A Meta-Analysis of Randomized Controlled Trials
Source: PLoS One. 2013 May 1;8(5):e62298. doi: 10.1371/journal.pone.0062298 (PMC3641077; doi:10.1371/journal.pone.0062298)

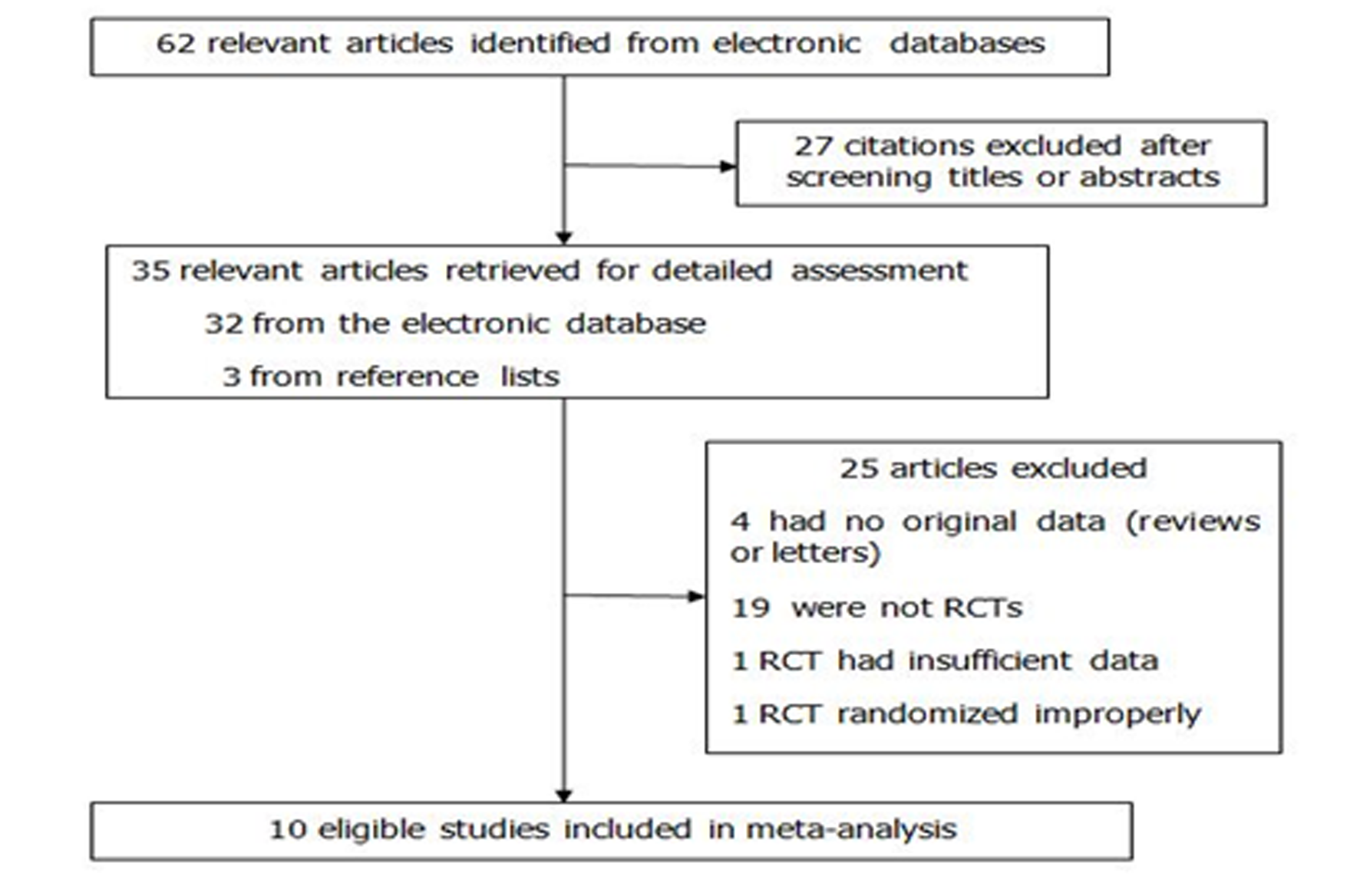

Supplement: Figure S1 — Flow diagram of the literature search. (TIF) [file pone.0062298.s001.tif]

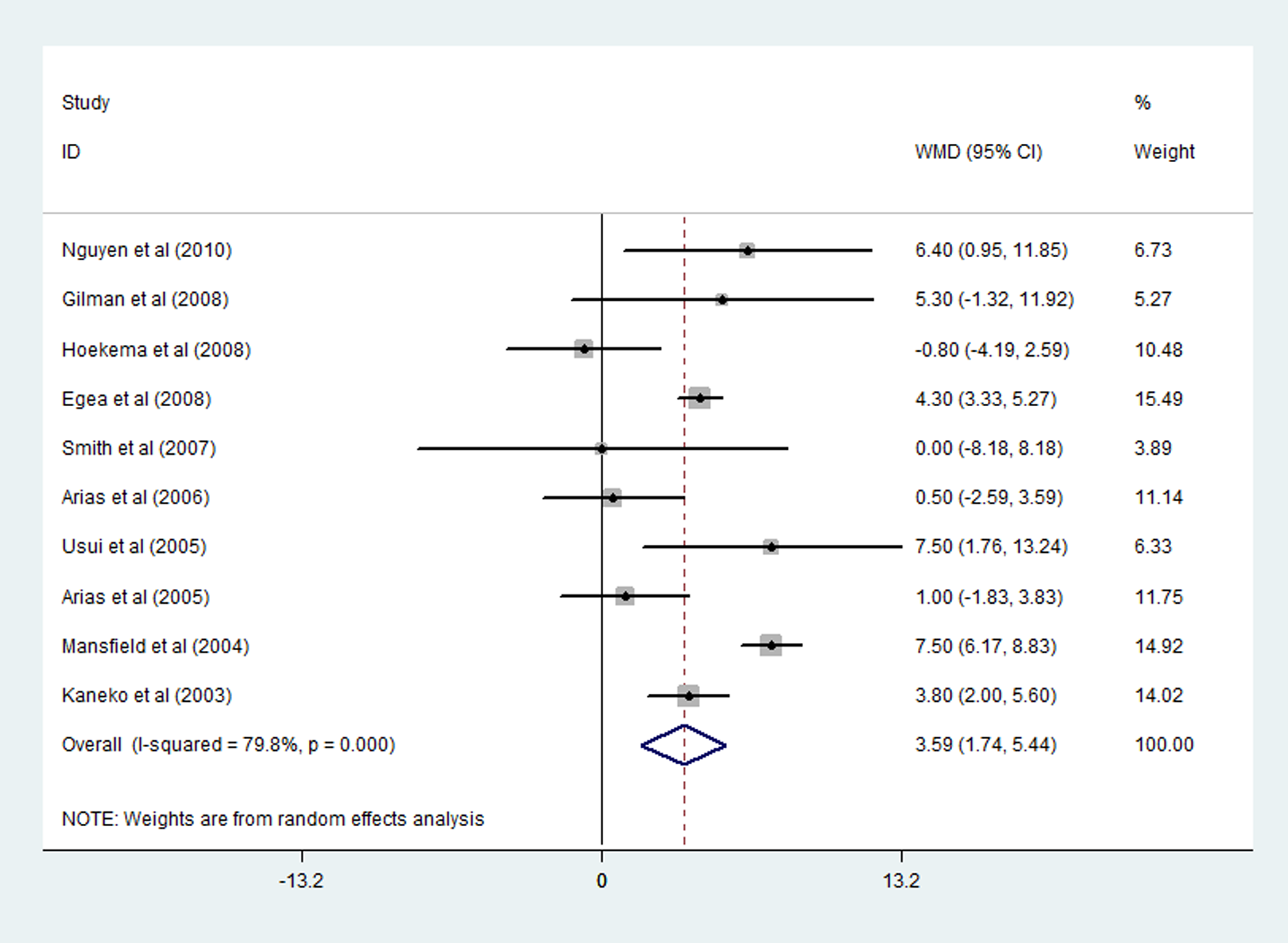

Supplement: Figure S2 — Forest plot presenting the meta-analysis for the effect of CPAP treatment on LVEF. WMD: weighted mean difference; CI: confidence intervals. (TIF) [file pone.0062298.s002.tif]

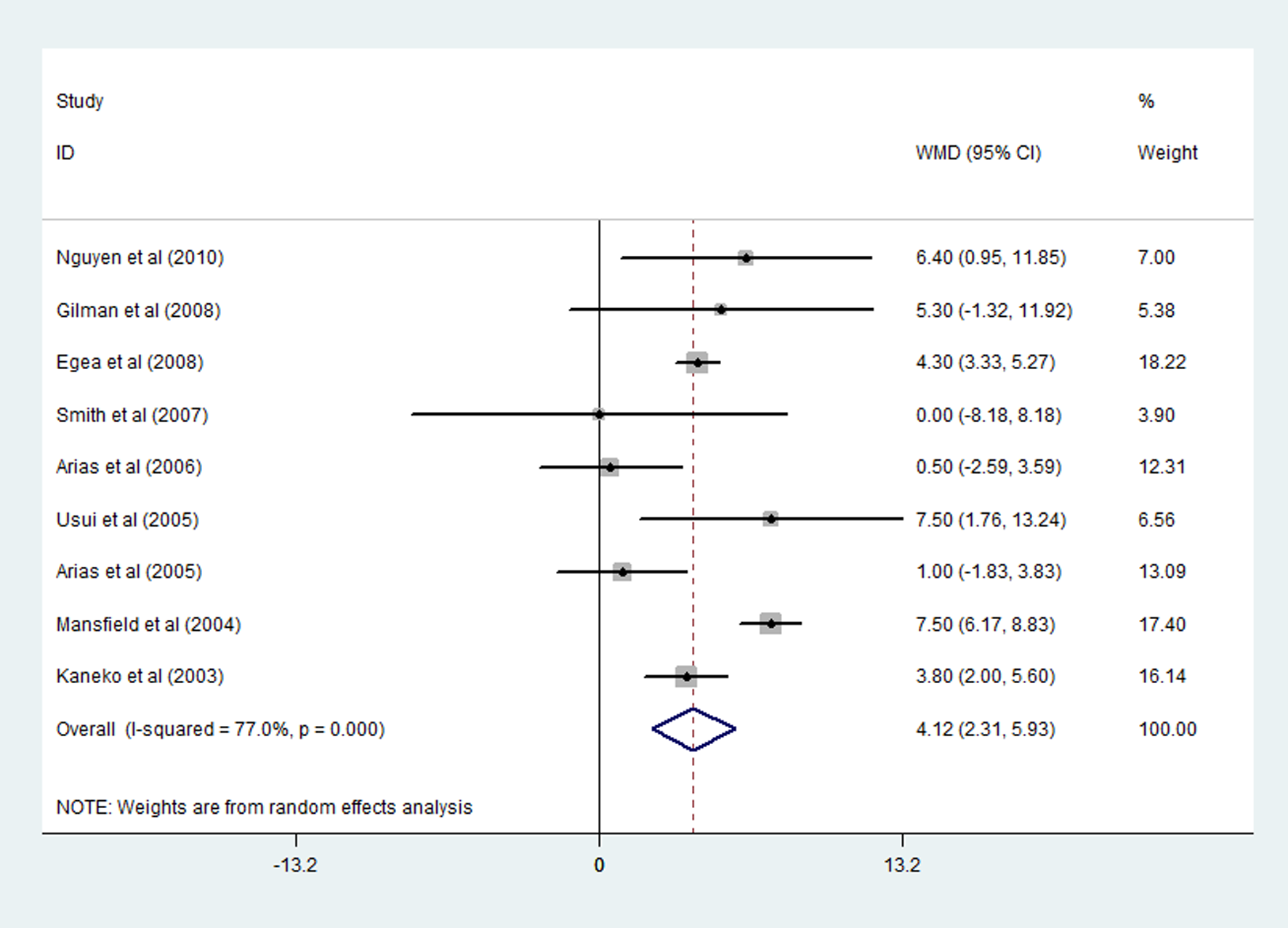

Supplement: Figure S3 — Forest plot presenting the meta-analysis for the effect of CPAP treatment on LVEF except Hoekema’s study. WMD: weighted mean difference; CI: confidence intervals. (TIF) [file pone.0062298.s003.tif]

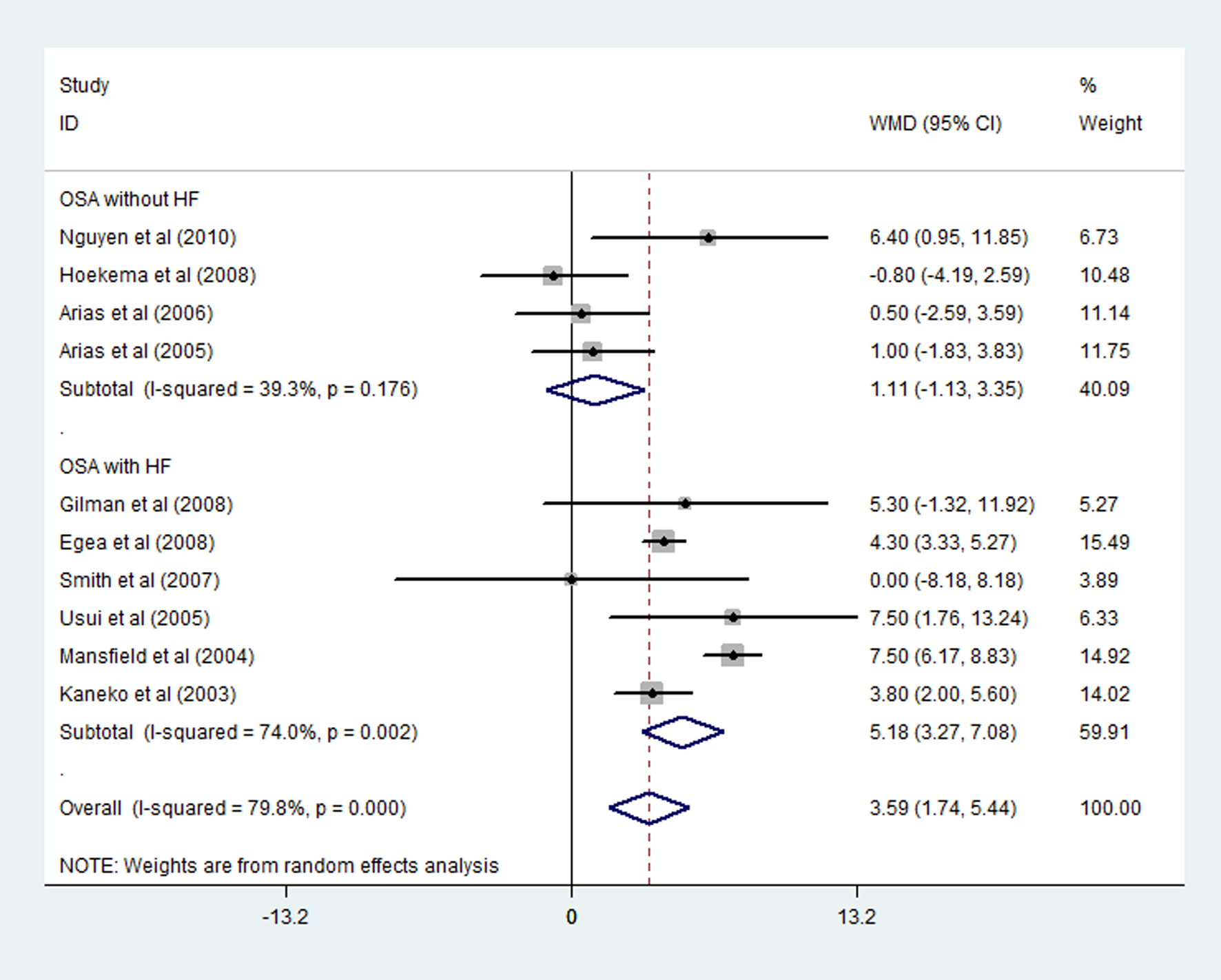

Supplement: Figure S4 — Forest plot presenting the meta-analysis for the effect of CPAP treatment on LVEF which were categorized by different patients. WMD: weighted mean difference; CI: confidence intervals. (TIF) [file pone.0062298.s004.tif]

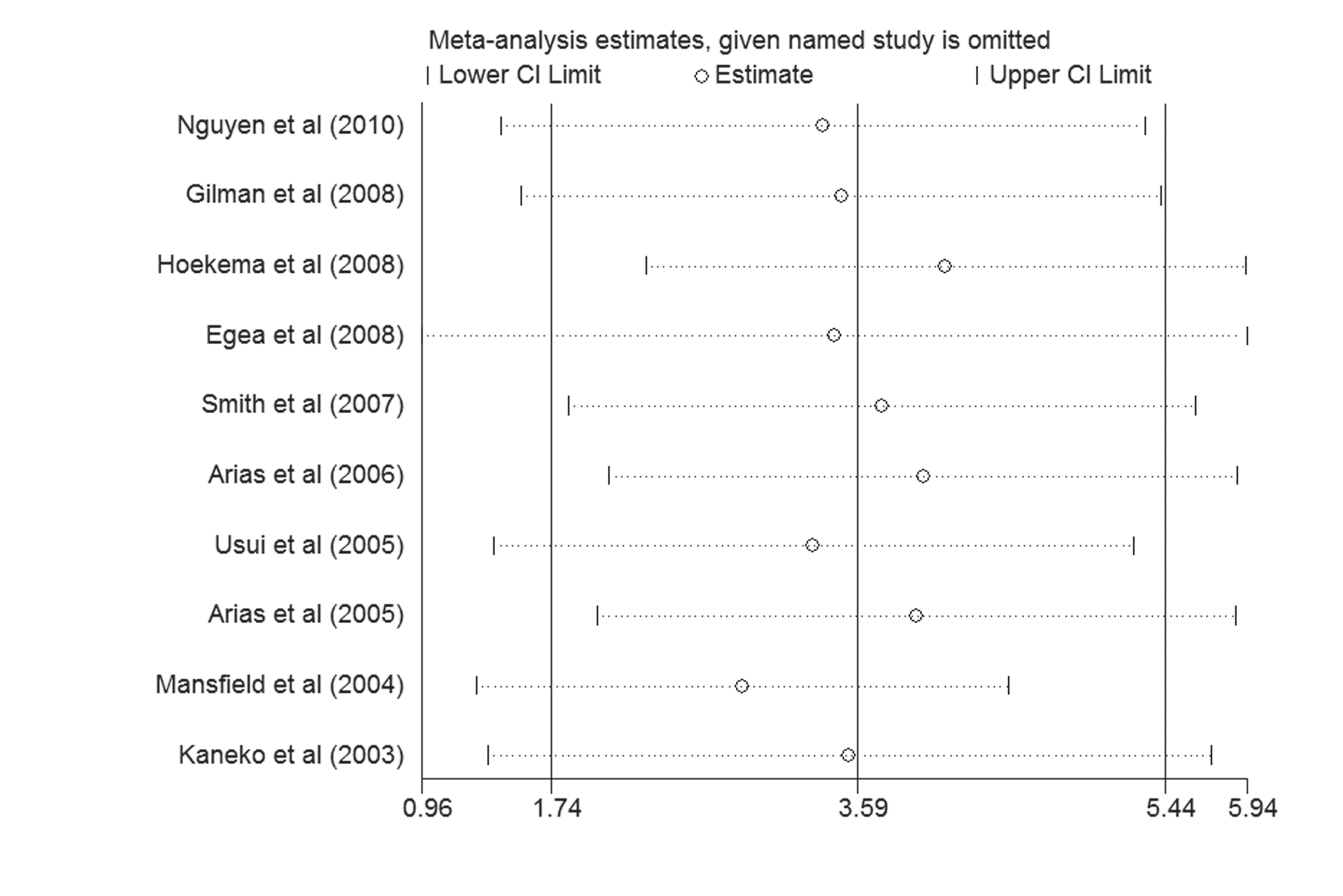

Supplement: Figure S5 — Sensitivity analysis of selected studies. CI: confidence intervals. (TIF) [file pone.0062298.s005.tif]
